# Evolution of a Potential Hormone Antagonist following Gene Splicing during Primate Evolution

**DOI:** 10.1371/journal.pone.0064610

**Published:** 2013-05-28

**Authors:** Cheng Deng, Aaron J. W. Hsueh

**Affiliations:** Program of Reproductive and Stem Cell Biology, Department of Obstetrics/Gynecology, Stanford University School of Medicine, Stanford, California, United States of America; University College London, United Kingdom

## Abstract

Alternative splicing of genes generates novel mRNAs, leading to the evolution of new functional proteins. Cholecystokinin (CCK) induces the release of pancreatic enzymes and the contraction of the gallbladder to promote the digestion of fat and proteins. CCK activates two G-protein-coupled receptors, CCKA and CCKB. Here, we showed that a CCKsv (splicing variant), originated de novo during Catarrhini evolution by including a portion of intronic sequence of the CCK gene, encodes novel C-terminal peptide sequence followed by a new poly-adenylation signal. CCKsv is expressed in many human tissues and likely a secreted peptide retaining the original signal peptide and the N-terminal proteolytic processing signal, together with novel C-terminal sequences. Although CCKsv cannot activate CCK receptors, it partially inhibits the CRE- or SRF-driven reporter activities stimulated by wide type CCK-8 mediated by both CCK receptors. Co-treatment with CCKsv also partially antagonizes Ewing tumor cell growth stimulated by CCK-8. Our study provides an example of new peptide hormone antagonist evolution in primates.

## Introduction

Alternative splicing allows the generation of various gene products with different functions from a single gene and is a major mechanism of generating protein diversity in eukaryotes [1]–[5]. At least half, or more, of mammalian genes can be alternatively spliced [1], [2]. Thus, alternative splicing emerges as a major mechanism of generating protein diversity and splicing of coding regions in peptide hormone genes often results in additional protein domains [1]. Calcitonin and calcitonin-related polypeptide represent the classical example of novel hormone evolution from the same gene and both splicing variants [6] encode peptide hormones capable of binding and activating the same receptors (calcitonin receptor and calcitonin receptor-like) [7]. Likewise, a splicing variant of the GALP (galanin-like peptide) gene generates a novel vasoactive peptide, alarin, without a known receptor [8], [9]. With the completion of the sequencing of more genomes from different vertebrates and transcriptomes from diverse tissues, a more complete picture of alternative splicing events start to emerge. Cholecystokinin (CCK), one of the first gastrointestinal hormones discovered, is a brain/gut peptide found to induce the release of pancreatic enzymes and the contraction of the gallbladder [10]–[12]. Here, we identified a splicing variant of the human CCK gene, encoding a new peptide hormone, CCKsv (splicing variant). CCKsv emerged during Catarrhini evolution and is a potential antagonist for CCK peptide.

## Materials and Methods

### Materials

HEK293T, KATO III, SK-PN-DW, and SK-N-MC cell lines were purchased from American Type Culture Collection. CCK-8 peptide was purchased from Phoenix Pharmaceuticals Inc. (Burlingame, CA) whereas CCKsv (GKNAASPSLTSALVPRLPMLTLFSSASLMGMTSL-amidated) was synthesized by the PAN facility at Stanford University. CRE, SRE, NFAT, SRF-luciferase reporter plasmids and the pSV-β-galactosidase control vector were purchased from the Promega.

### RT-PCR and CCKsv Cloning

Total RNAs from different cells were isolated using a RNA extraction kit (Qiagen) and eluted with RNase-free, DEPC-treated water before treatment with DNase. After reverse transcription using Sensiscript RT kit (Qiagen), the expression of CCK and CCKsv were analyzed using specific primers (Supplementary Fig. S2). The open reading frame of CCKsv were subcloned into the pcDNA3.1 plasmid and verified by DNA sequencing.

### Quantitative RT-PCR

Human normal cDNA Array for 48 tissue was brought from OriGene Technologies as the template. Quantitative PCR were performed in triplicates using iTaq SYBR Green Supermix kit (Bio-Rad) using primer sets shown in Supplementary Fig. S2. Expression levels for CCKsv were normalized based on GAPDH (Glyceraldehyde 3-phosphate dehydrogenase) levels.

### Secretion Analysis of CCKsv

V5-tagged CCKsv was cloned using specific primers (Supplementary Fig. S2) into the template CCKsv cDNA and the full V5 tagged CCKsv protein sequence is shown in Supplementary Fig. S4. The SK-N-MC cells were transfected with V5-tagged CCKsv or empty vector. One day later, cells were treated with serum-free media for another 3 days. After concentration, the media was used for immunoblotting analysis with the anti-v5 antibody (Cell Signaling) and the cell lysate for immunoblotting analysis with the anti-GAPDH antibody (Cell Signaling), respectively.

### Luciferase Assays

HEK293T cells seeded in 24-well plates were co-transfected by various luciferase reporters (30 ng), the pSV-β-Gal plasmid (3 ng), and the CCKA or CCKB receptor (30 ng). After 1 day, cells were treated in serum-free media for another 18 h with increasing doses of the CCK-8 or CCKsv peptide. For the estimation of antagonistic activities, cells were pre-incubated (30 min.) with the CCKsv peptide before treatment with CCK8 for 18 h. Luciferase activities were determined using luciferase assay kits (Promega) and normalized using β-galactosidase activities. All experiments were performed at least three times in triplicates. Data were analyzed using Graphpad Prism 5.0.

### In vitro Ewing Tumor Cell Growth Studies with CCK-8 and CCKsv Peptides

SK-N-MC and SK-PN-DW cells (1×10^5^) were plated in 12-well plates and allowed to attach for 24–36 h. Cells were then cultured in serum-free media with 0.1 uM of the CCK-8 peptide with or without the CCKsv peptide. Fresh media containing peptides were replaced every two days. To demonstrate CCKsv antagonism, cells were pre-treated with different dose of CCKsv for 30 min. before adding the CCK-8 peptide. Cell growth was determined by crystal violet staining [13].

### Statistics

Experiments were repeated independently at least 3 times. Calculations were done with a standard statistical package (SPSS for Windows, version 21). Statistical significance was defined as a *P* value <0.05 (*) and *P* value <0.01(**).

## Results

**Figure 1:**
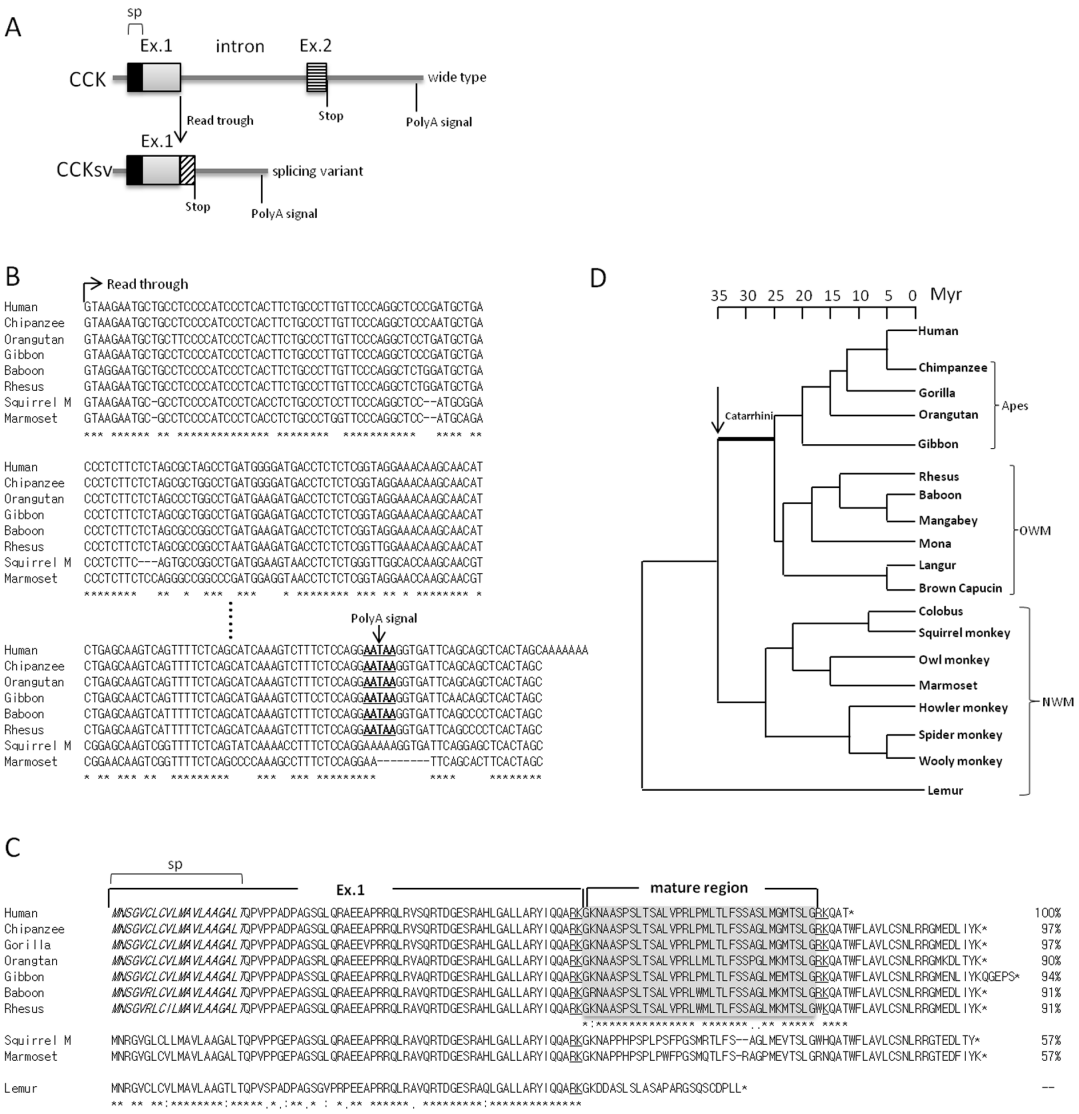
CCKsv originated by including a portion of intronic sequence of the CCK gene and evolved in the Catarrhini lineage. (**A**), Gene structure of CCK and CCKsv. Human CCK gene has two exons and one intron. Wild type CCK protein is encoded by exon1 and exon2 with exon1 containing the signal peptide (sp) and exon2 containing the mature peptide region (stripped bar). CCKsv is encoded by exon1, followed by a read through into the intron region, leading to the generation of novel mature region (hatched bar), followed by a stop codon and a new poly-adenylation signal. (**B**), Alignment of CCKsv read though sequences in diverse primate species. Sequences encoding the putative poly-adenylation signal are in bold and underlined. Full nucleotide sequence alignment is shown in Supplementary Fig. S7. (**C**), Alignment of predicted CCKsv protein sequences in primates. Genomic DNA sequences found in NCBI and UCSC genome databases were translated and deduced protein sequences aligned by the ClustalW program [36]. Signal peptide (sp) is in italic. Putative mature regions of CCKsv were shaded and proteolytic processing signal underlined. Similarity to the human mature peptide region is shown as percentages on the right side. (**D**), Phylogenetic tree of the primate species. Phylogenetic relationships among different primate species are based on previous studies [37], [38] with the divergence times [35] indicated. An arrow denotes the time when the CCKsv emerged.

One complete cDNA (GenBank sequence ID: AK300784) representing an alternative splicing form of the CCK mRNA was found in a cDNA library of a human neuronal epithelioma Ewing tumor cell line. Based on the analysis of genome sequences of the human CCK gene (Fig. 1A), wild type CCK is derived from exon1 encoding a signal peptide together with exon2 encoding the mature region of the mature CCK peptide, followed by a stop codon and a poly-adenylation signal. In contrast, the CCKsv retains the entire exon1 with a read through into the original intron sequence followed by a new stop codon, thus deriving a novel C-terminus of the coding region and a new poly-adenylation signal (Fig. 1A).

To trace the origination of CCKsv, the syntenic chromosome locations of orthologous CCK genes in diverse primate species were identified, followed by the alignment of DNA sequences (Fig. 1B) and predicted protein sequences (Fig. 1C). Although wild type CCK coding regions in exon1 and exon2 were conserved in all primates (Supplementary Fig. S1), a putative novel poly-adenylation signal was found to be conserved in the intron of human, three apes, and two old world monkeys (Fig. 1B). However, this region was not found in new world monkeys (squirrel monkey and mamorset) (Fig. 1B). Based on deduced protein sequences of the new mature peptide region (Fig. 1C), the novel human CCKsv sequence is highly conserved (>90%) in human, four apes, and two old world monkeys. However, no poly-adenylation signal was found in new world monkeys (Fig. 1B). In addition, virtual translation of the corresponding regions in new world monkeys indicated less than 60% conservation with human CCKsv (Fig. 1C). Many peptide hormones have a typical amidation/proteolytic processing signal at the C-terminal end characterized by a glycine residue followed by two basic amino acids, thus generating amidated peptides more resistant to proteolytic degradation [14]. Like the amidated wild type CCK (Supplementary Fig. S1), comparison of CCKsv sequences in Catarrhini indicated the conservation of a glycine residue followed by two basic residues (Fig. 1C). In contrast, this amidation/proteolytic processing signal was not found in new world monkeys (Fig. 1C). Based on phylogenetic analyses of different primate species, one can conclude that CCKsv originated de novo by including a portion of intronic sequence of the CCK gene in the common ancestor of Catarrhini primates (Fig. 1D).

**Figure 2:**
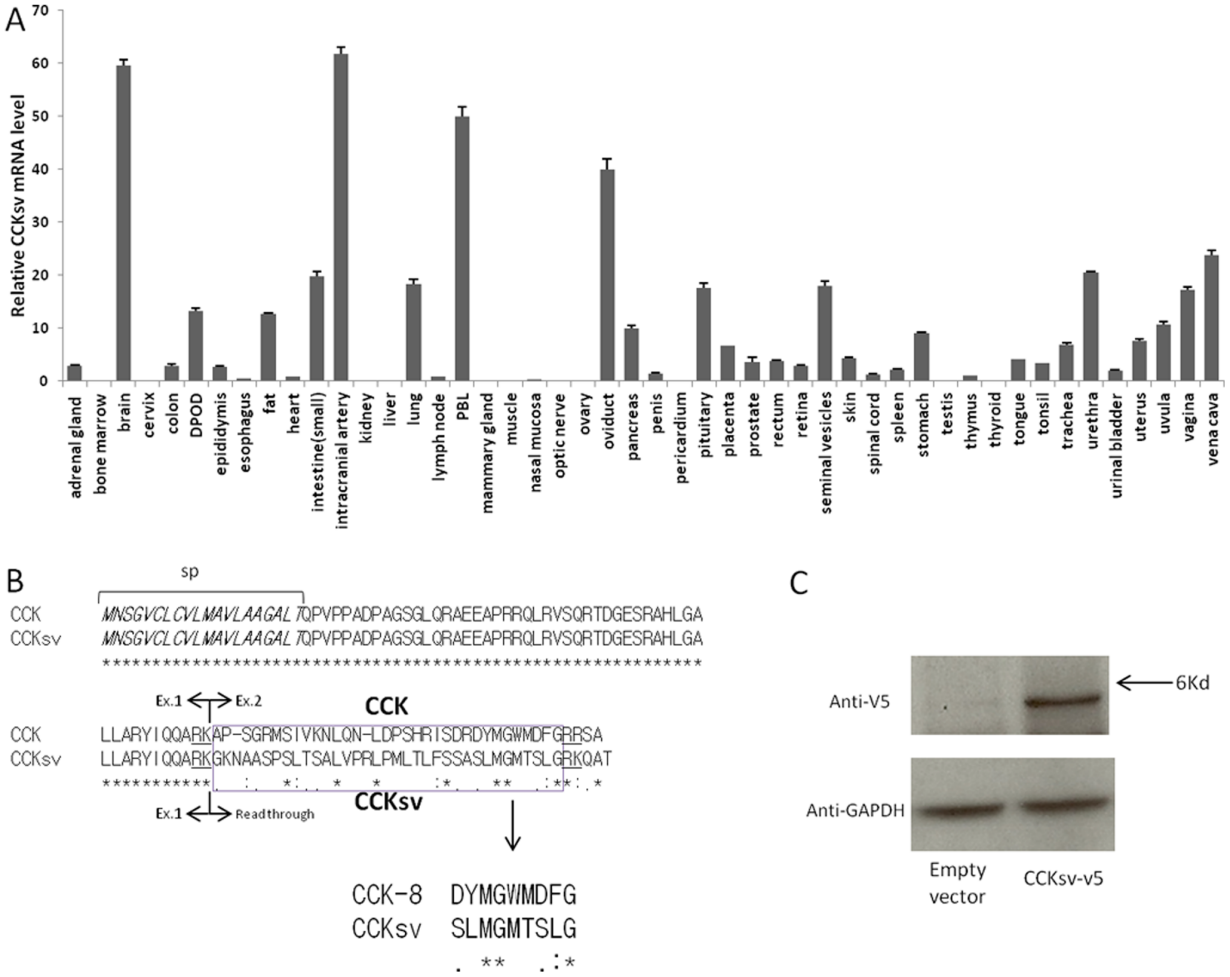
CCKsv is expressed in diverse tissues and encodes a secreted peptide. (**A**) Expression of CCKsv mRNAs in different human tissues. Quantitative RT-PCR was carried out using Human Normal cDNA Array (Origene) as the template together with specific primer sets (Supplementary Fig. S2). Expression of CCKsv mRNAs were normalized based on GAPDH levels. Human tissues include adrenal gland, bone marrow, brain cervix, colon, DPOD (descending part of duodenum), epididymis, esophagus, fat, heart, small intestine, intracranial artery, kidney, liver, lung, lymph node, PBL (plasma blood leucocytes), mammary gland, muscle, nasal mucosa, optic nerve, ovary, oviduct, pancreas, penis, pericardium, pituitary, placenta, prostate, rectum, retina, seminal vesicles, skin, spinal cord, spleen, stomach, testis, thymus, thyroid, tongue, tonsil, trachea, urethra, urinal bladder, uterus, uvula, vagina, and vena cava. (**B**), Alignment of protein sequences of CCK and CCKsv. Signal peptide is in italic. Similar mature regions of CCK and CCKsv are boxed and the proteolytic processing signal underlined. (**C**), Secretion analysis of CCKsv. SK-N-MC cells were transfected with V5-tagged CCKsv or an empty vector. One day later, cells were treated with serum-free media for another 3 days. After concentration, the media were used for immunoblotting analysis using the anti-v5 antibody and the cell lysate analyzed using the anti-GAPDH antibody.

The expression pattern of human CCKsv in diverse human tissues was analyzed by quantitative RT-PCR using specific primers (Supplementary Fig. S2). Like wild-type CCK [15], CCKsv was found in diverse human tissues, with higher expression in brain, intracranial artery, plasma blood leucocytes, and oviduct (Fig. 2A). The expression of CCKsv, together with wild type CCK, was further confirmed by RT-PCR in human SK-N-MC and stomach carcinoma KATO-III cells (Supplementary Fig. S3). No expression was found in HEK 293T cells (Supplementary Fig. S3).

The coding sequence of CCKsv was compared with that of the wild type CCK (Fig. 2B). CCKsv lost the entire CCK mature peptide region (Fig. 2B, read through) but still retained the signal peptide and N- terminal proteolytic processing signal “RK” encoded by exon1, followed a novel peptide sequence. A new conserved C-terminal end “GRK” motif, presumably important for proteolytic processing, was found (Fig. 1C), leading to a predicted mature peptide with a moderate similarity to wild type CCK (Fig. 2B). Wild type CCK protein can be processed into at least four variants (CCK-58, CCK-33, CCK-22 and CCK-8) with a common C-terminal end of eight residues [12]. All these peptide showed similar agonistic activities at the two CCK receptors [16], [17], indicating the important role of the C-terminal region for receptor recognition. When the C-terminal ends of CCK and CCKsv were aligned, 5 out of eight residues were similar (Fig. 2B), suggesting CCKsv could also interact with CCK receptors.

To demonstrate that the novel CCK splicing transcript encodes a new peptide hormone that could be processed by proteolytic enzymes and secreted, we cloned the full length CCKsv cDNA from human SK-N-MC cells and inserted a V5 epitope tag (Supplementary Fig. S4) for expression analysis in transfected SK-N-MC cells. As showed in Fig. 2C, immunoblotting analysis indicated a positive signal at ∼5 kd using the V5 antibody in the conditioned media, suggesting the putative CCKsv peptide could be processed and secreted (Fig. 2B).

**Figure 3:**
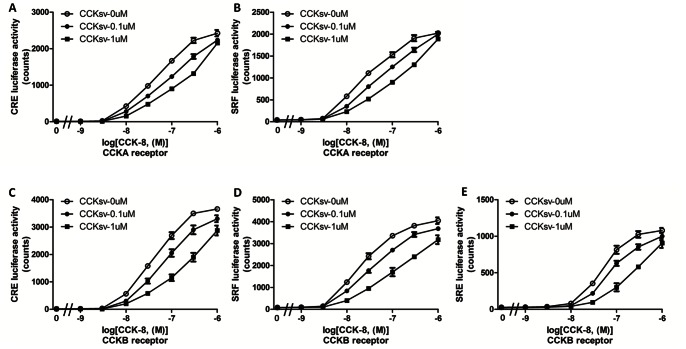
CCKsv antagonized CCK activity mediated by CCKA and CCKB receptors. Stimulations of CRE (**A**) or SRF (**B**) luciferase activities by CCK-8 peptide mediated by the CCKA receptor were inhibited by the CCKsv peptide. In addition, CCK-8-stimulated CRE (**C**), SRF (**D**), or SRE (**E**) luciferase activities mediated by the CCKB receptor were also inhibited by the CCKsv peptide. HEK293T cells were co-transfected by CRE-, SRE-, or SRF-luciferase reporters, together with the pSV-β-galactosidase plasmid, and CCKA or CCKB receptors. After 1 day of culture, cells were pre-incubated (30 min.) with the CCKsv peptide (0.1 uM or 1 uM) before treatment with increasing doses of CCK-8 peptide. Data were analyzed using Graphpad Prism 5.0.

Wild type CCK binds CCKA and CCKB receptors to activate several signaling pathways mediated by G proteins, including the adenyl cyclase and phospholipase C-diacylglycerol-protein kinase C pathways [18]. CCK also induces the mitogen-activated protein (MAP) kinase pathway mediated by ERK, JNK, and p38-MAPK [17]. Different G proteins (Gs, Gi, Gq, and G12) activated by diverse GPCRs can be analyzed using luciferase reporters containing different response elements, CRE-, SRE-, NFAT-, and SRF-RE [19], [20]. We screened diverse reporter systems using HEK293T cells and found that the CCK-8 peptide stimulated CRE- and SRF-RE reporters via CCKA after 18 h of incubation whereas CCKB receptor mediated the stimulation of CRE-, SRE- and SRF-RE reporters by the CCK-8 peptide (Fig. 3). Because the CCKsv mature region shares moderate similarity with the wild type CCK mature peptide, we further checked if CCKsv can activate any G protein pathway via CCKA and B receptors. As shown in Supplementary Fig. S5, CCksv is not capable of stimulating different G proteins (Gs, Gi, Gq, and G12)-mediated by the CCKA or the CCKB receptor based on the monitoring of CRE-, SRE-, NFAT-, SRF-RE reporter assays [19], [20], thus ruling out its agonistic activities at the CCK receptors. Peptide hormone antagonists often have sequence similarity with critical domains as the original peptide, allowing them to bind but not activate specific receptors [21]. Because CCKsv showed sequence homology with the C-terminal end of wild type CCK (Fig. 2B) and could be secreted and processed in SK-N-MC cells (Fig. 2C), we chemically synthesized the CCKsv peptide to test its potential antagonist functions. HEK293T cells transfected with CCKA or CCKB receptor, together with different luciferase receptors, were pre-incubated with CCKsv for 30 min. before treatment with various doses of CCK-8 peptide for another 18 h. As shown in Fig. 3, cells pretreated with CCKsv displayed a dose-dependent suppression of CCK8 actions, suggesting CCKsv is a potential antagonist for CCK. Cells overexpressing the CRE reporter and the CCKB receptor were most responsive to CCK-8 peptide stimulation (Fig. 3C). In these cells, 1 uM of CCKsv suppressed CCK-8 peptide (30 nM) stimulated CRE and SRF luciferase activity by ∼60% (Fig. 3C and D). To rule out non-specific effects of CCKsv, we further checked if co-treatment with CCKsv inhibits the luciferase activity stimulated by relaxin in LGR7-expressing cells [22]. As shown in Supplementary Fig. S6, CCKsv (0.1 uM or 1 uM) did not alter relaxin signaling to rule out its toxic effect.

**Figure 4:**
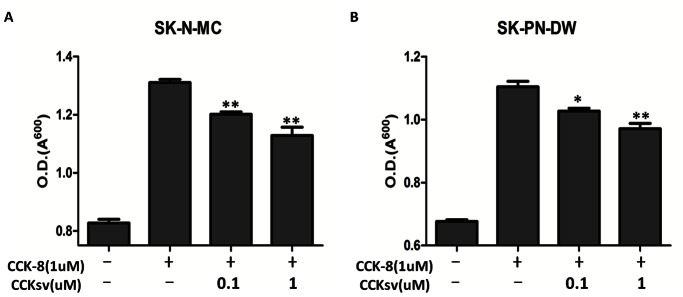
CCKsv peptide inhibits the Ewing tumor cell growth stimulated by CCK-8 peptide. SK-N-MC and SK-PN-DW Ewing tumor cells were plated in 12-well plates and treated with 0.1 uM of the CCK-8 peptide. Fresh serum-free media with CCK-8 peptide were replaced every two days. To test CCKsv antagonism, cells were pre-treated with different doses (0.1 uM and 1 uM) of CCKsv for 30 min. before adding the CCK-8 peptide. After 6 days of treatment, cell growth was determined by crystal violet staining. (**A**), SK-N-MC cells; (**B**), SK-PN-DW cells. *P<0.05. **P<0.01.

CCK induces cell proliferation in various cancer cell lines [23]–[27] and serves as an autocrine growth factor in Ewing tumor cells [13]. Because treatment with a CCK antagonist inhibits proliferation of Ewing tumor cells, CCK antagonist could represent a new therapeutic approach in the management of Ewing's tumor patients [28]. To further confirm the potential antagonist function of CCKsv, we checked if CCKsv could inhibit Ewing tumor cell growth stimulated by CCK. Two Ewing tumor cell lines (SK-N-MC and SK-PN-DW) were cultured with the CCK-8 peptide (0.1 uM) in the presence or absence of CCKsv (0.1 uM or 1 uM). After 6 days, cell growth was quantified using crystal violet staining. As shown in Fig. 4, co-treatment with CCKsv dose-dependently reduced CCK-stimulated growth of both SK-N-MC and SK-PN-DW cells. In SK-N-MC cell line, co-treatment with 0.1 and 1 uM CCKsv suppressed CCK actions by 22 and 38%, respectively (Fig. 4A). Likewise, co-treatment with CCKsv suppressed CCK actions in SK-PN-DW cells by 18 and 31%, respectively (Fig. 4B). The results again showed the CCKsv can partially antagonize CCK stimulation of cell growth. Further analyses of structural-functional relationship of CCKsv could allow the development of candidate drugs for cancer therapy.

## Discussion

Peptide hormones and their receptors often show high conservation in vertebrates, even during metazoan evolution [29], thus making the tracing of their origination difficult. Alternatively processed calcitonin and calcitonin-related polypeptide share the similar mature peptide structure probably due to exon duplication and rearrangement in vertebrates [6]. In addition, alarin originated from the GALP gene and showed conservation of the first 10 residues between rodents and primates, thus likely originated during Euarchontoglires evolution [8], [9]. Even though alarin was found to regulate vascular functions [8] and stimulate food intake [30], [31], its receptor is still unclear. Although more than 20 splicing variants of the gene encoding the gastrointestinal peptide ghrelin have been identified in different human tissues [32], their secretion and function remain unclear. Also, splicing variants for the VIP (Vasoactive intestinal peptide) gene was found in chicken and turkey but not mammals [33], [34]. As shown in Fig. 1B and C, CCKsv from all Catarrhini species showed conserved novel C-terminal mature regions, amidation/proteolytic processing signal, and poly-adenylation signals. The newly acquired poly-adenylation signal (AATAA) was found in human, all apes and old world monkeys but missing in the two available new world monkey sequences (AAAAA for squarrel monkey and AA– for marmoset) (Fig. 1B). These features of CCKsv likely originated during early Catarrhini evolution about 35 million years ago when old world monkeys (Catarrhini) diverged from the new world monkeys (Platyrrhini) (Fig. 1D) [35]. Alternatively, one cannot rule out the possibility that CCKsv was lost in the lineage leading to the common ancestor of Squirrel monkey and Marmoset.

Based on the high conservation of CCKsv in the Catarrhini lineage, this young peptide is likely under selection and its function could still be evolving. Of interest, novel stop codons were found in human and Gibbon sequences (Fig. 1C). Because these stop codons were behind the convertase cleavage site (GRK), the mature CCKsv sequences for these two species remain the same (Fig. 1C). CCK is known to be important for Ewing tumor progression [13], further studies on the antagonistic functions of CCKsv and its derivatives could allow the formulation of new peptides for anticancer therapy.

## Supporting Information

Figure S1 **Alignment of wild type CCK protein sequences in primates.** Signal peptide is in italic. Mature regions of CCKsv were shaded and proteolytic processing signal underlined. Arrow showed the glycine residue important for amidation.
(PDF)

Figure S2 **CCKsv expression analyses in cell lines.** Total RNAs isolated from different cells were used as PCR templates to avoid genomic DNA contamination in total RNA. Each band for CCK and CCKsv were sub-cloned and sequenced for confirmation.
(PDF)

Figure S3 **PCR primers sets.**
(PDF)

Figure S4 **Protein sequence of V5 tagged CCKsv.** The V5 tag was inserted into the CCKsv mature region.
(PDF)

Figure S5 **CCKsv does not stimulate diverse G protein signaling in CCKA or CCKB receptor expressing HEK293T cells.** (A) CRE-luciferase for Gs activity, (B) SRE-luciferase for Gi and Gq activities, (C) NFAT-luciferase for Gq activity, (D) SRF-RE-luciferase for G12 activity.
(PDF)

Figure S6 **CCKsv cannot alter the CRE-luciferase activity stimulated by relaxin in LGR7-expressing cells.**
(PDF)

Figure S7 **Alignment of CCKsv read though sequences in primates.** The putative poly-adenylation signal is shown in bold and underlined.
(PDF)
