# Supplementary material for: Evolution of a Potential Hormone Antagonist following Gene Splicing during Primate Evolution
Source: PLoS One. 2013 May 28;8(5):e64610. doi: 10.1371/journal.pone.0064610 (PMC3665846; doi:10.1371/journal.pone.0064610)
Supplement: Figure S1 — Alignment of wild type CCK protein sequences in primates. Signal peptide is in italic. Mature regions of CCKsv were shaded and proteolytic processing signal underlined. Arrow showed the glycine residue important for amidation. (PDF) [file pone.0064610.s001.pdf]

Fig. S1

```
Human            MNSGVCLCVLMAVLAAGALTQPVPPADPAGSGLQRAEEAPRRQLRVSQRTDGESRAHLGA
Chipanzee        MNSGVCLCVLMAVLAAGALTQPVPPADPAGSGLQRAEEAPRRQLRVSQRTDGESRAHLGA
Orangutan        MNSGVCLCVLMAVLAAGALTQPVPPADPAGSRLQRAEEEPRRQLRVAQRTDGESRAHLGA
Gibbon           MNSGVCLCVLMAVLAAGALTQPVPPADPASSGLQRAEEAPRRQLRVAQRTDGESRAHLGA
Baboon           MNSGVRLCVLMAVLAAGALTQPVPPAEPAGSGLQRAEEAPRRQLRAVQRTDGESRAHLGA
Rhesus           MNSGVSLCVLMAVLAAGALTQPVPPAEPAGSGLQRAEEAPRRQLRAVQRTDGESRAHLGA
Squirrel_monkey  MNRGVGLCLLMAVLAAGALTQPVPPGEPAGSGLQRAEEAPRRQLRAVQRTDGESRAHLGA
Marmosets        MNRGVGLCVLMAVLAAGALTQPVPPGEPAGSGLQRAEEAPRRQLRAVQRTDGESRAHLGA
Otolemur         MNRGVCLCVLMAVLAAGTLTQPVSPADPAGSGVPRPEEAPRRQLRAVQRTDGESRAQLGA
                 ** ** **:********:*****.*.:**.* : *.** ******. *********:***

                         Ex.1 <-> Ex.2
Human            LLARYIQQARKAPSGRMSIVKNLQNLDPSHRISDRDYMGWMDFGRRSAEEYEYPSL
Chipanzee        LLARYIQQARKAPSGRMSVVKNLQNLDPSHRISDRDYMGWMDFGRRSAEEYEYPS-
Orangutan        LLARYIQQARKAPSGRMSIVKNLQNLDPSHRISDRDYMGWMDFGRRSAEEYEYPS-
Gibbon           LLARYIQQARKAPSGRMSIVKNLQNLDPSHRISDRDYMGWMDFGRRSAEEYEYPS-
Baboon           LLARYIQQARKAPSGRMSIIKNLQNLDPSHRISDRDYMGWMDFGRRSAEEYEYPS-
Rhesus           LLARYIQQARKAPSGRMSIIKNLQNLDPSHRISDRDYMGWMDFGRRSAEEYEYPS-
Squirrel_monkey  LLARYIQQARKAPSGRTPVIKNLQNLDPSHRISDRDYMGWMDFGRRSAEEYEYPS-
Marmosets        LLARYIQQARKAPSGRMSVVKNLQNLDPSHRISDRDYMGWMDFGRRSAEEYEYPS-
Otolemur         LLARYIQQARKAPSGRNSIIKNLQSLDPSHRISDRDYMGWMDFGRRSAEEYEYPS-
                 **************** .::****.******************************
```
