# Supplementary material for: Evolution of a Potential Hormone Antagonist following Gene Splicing during Primate Evolution
Source: PLoS One. 2013 May 28;8(5):e64610. doi: 10.1371/journal.pone.0064610 (PMC3665846; doi:10.1371/journal.pone.0064610)
Supplement: Figure S2 — CCKsv expression analyses in cell lines. Total RNAs isolated from different cells were used as PCR templates to avoid genomic DNA contamination in total RNA. Each band for CCK and CCKsv were sub-cloned and sequenced for confirmation. (PDF) [file pone.0064610.s002.pdf]

Fig. S2

Name	sequence
ccksv QRT-PCR primer	
ccksv-fqpcr	TCACTTCTGCCCTTGTTC
ccksv-rqpcr	GTTGCTTGTTTCCTACCG
actin QRT-PCR primer	
GAPDH-F	AGAAGGCTGGGGCTCATTTG
GAPDH-R	AGGGGCCATCCACAGTCTTC
ccksv cloning primer	
CcksvATG	ATGAACAGCGGCGTGTGCCTGTG
CcksvTGA	TCATGTTGCTTGTTTCCTACCGA
cck cloning primer	
CckATG	ATGAACAGCGGCGTGTGCCTGTG
cckTAG	CTAGGAGGGGTACTCATACTCCT
V5 tagged ccksv cloning primer	
CcksvATG	ATGAACAGCGGCGTGTGCCTGTG
V5-tagged CcksvTGA	TCATGTTGCTTGTTTCCTACCGAGAGAGGTCATCCCCATCAGGCTAGCG CTAGAGAAGAGGGTCAGCATCGGGAGCGTAGAATCGAGACCGAGGA GAGGGTTAGGGATAGGCTTACCCCTGGGAACAAGGGCAGAAGTGAG GG
