# Supplementary figures and images for: Evolution of a Potential Hormone Antagonist following Gene Splicing during Primate Evolution
Source: PLoS One. 2013 May 28;8(5):e64610. doi: 10.1371/journal.pone.0064610 (PMC3665846; doi:10.1371/journal.pone.0064610)

Fig. S3

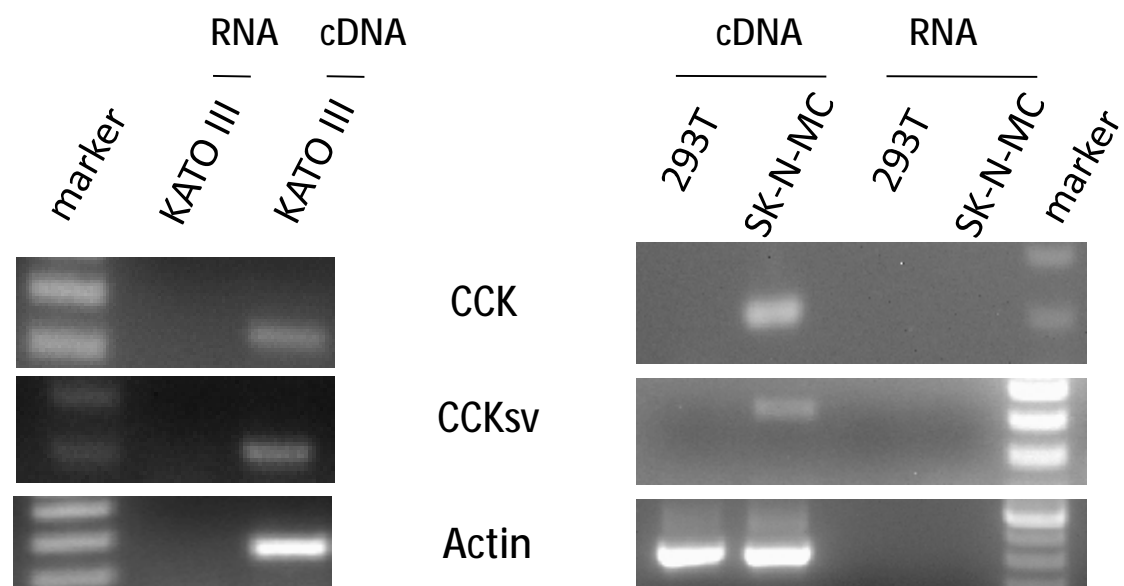

Supplement: Figure S3 — PCR primers sets. (PDF) [file pone.0064610.s003.pdf]

Fig. S5

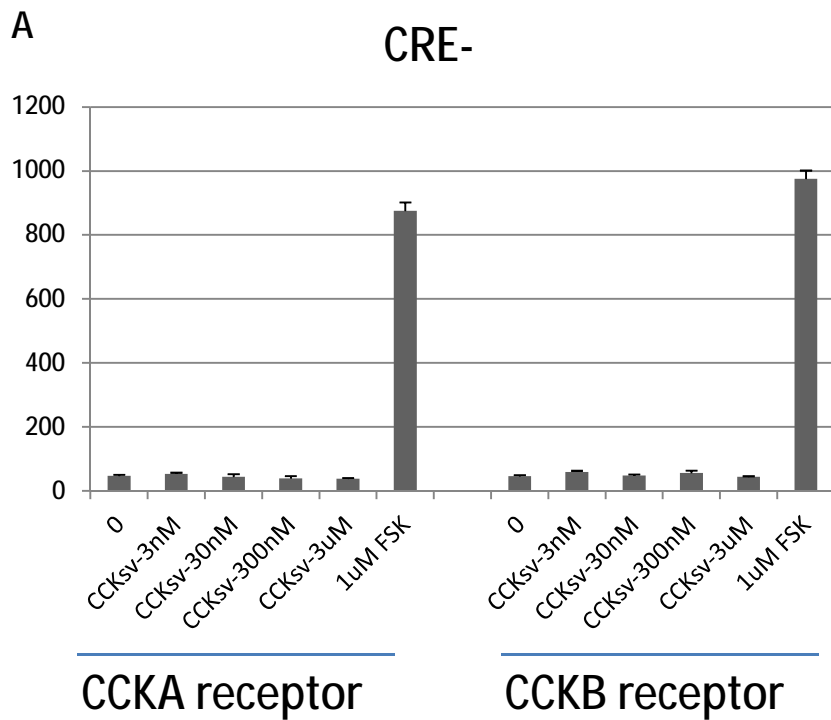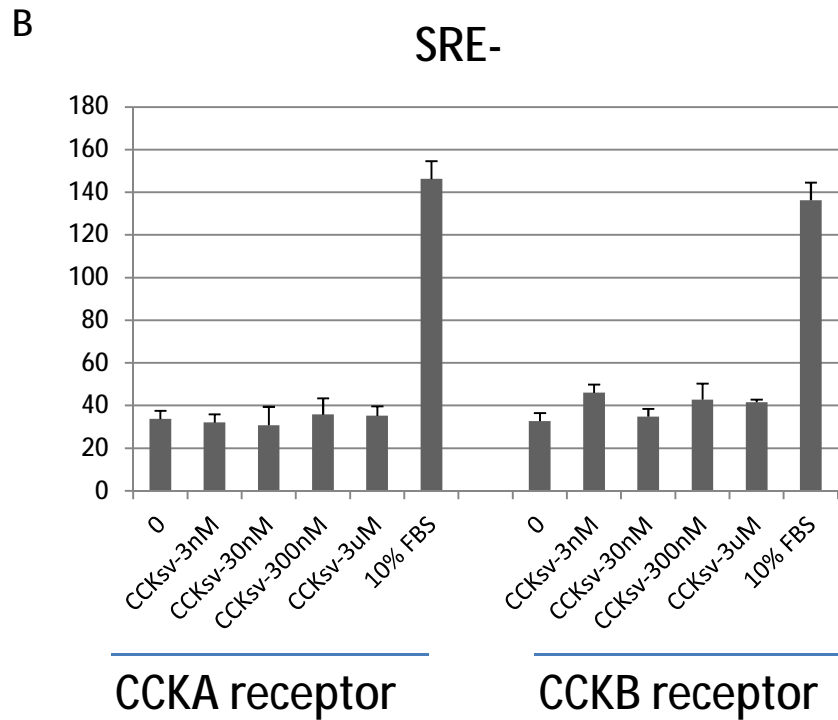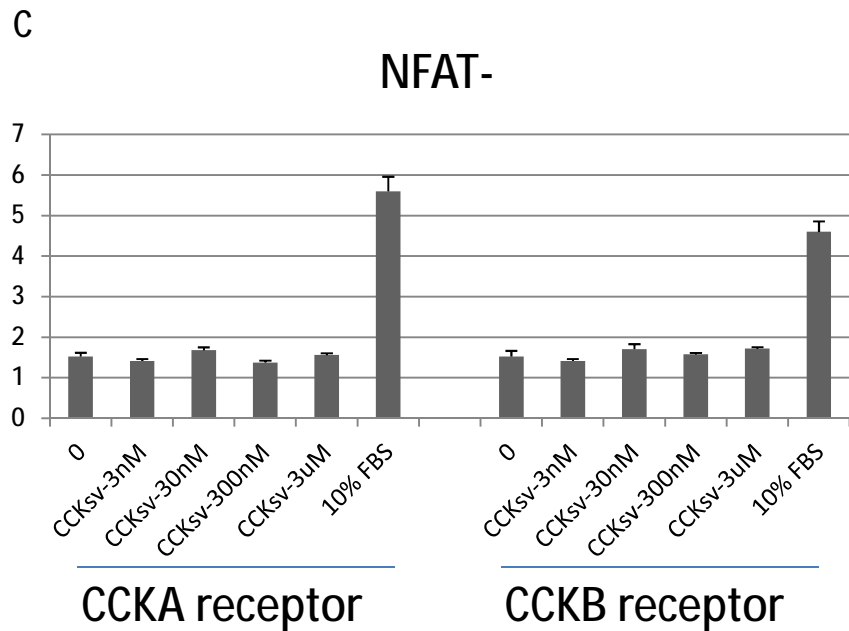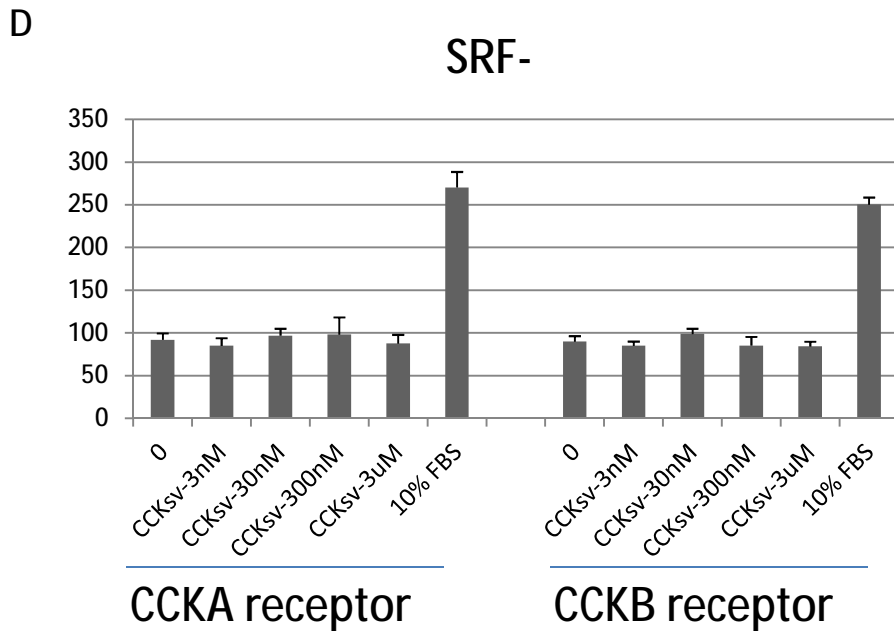

Supplement: Figure S5 — CCKsv does not stimulate diverse G protein signaling in CCKA or CCKB receptor expressing HEK293T cells. (A) CRE-luciferase for Gs activity, (B) SRE-luciferase for Gi and Gq activities, (C) NFAT-luciferase for Gq activity, (D) SRF-RE-luciferase for G12 activity. (PDF) [file pone.0064610.s005.pdf]

Fig. S6

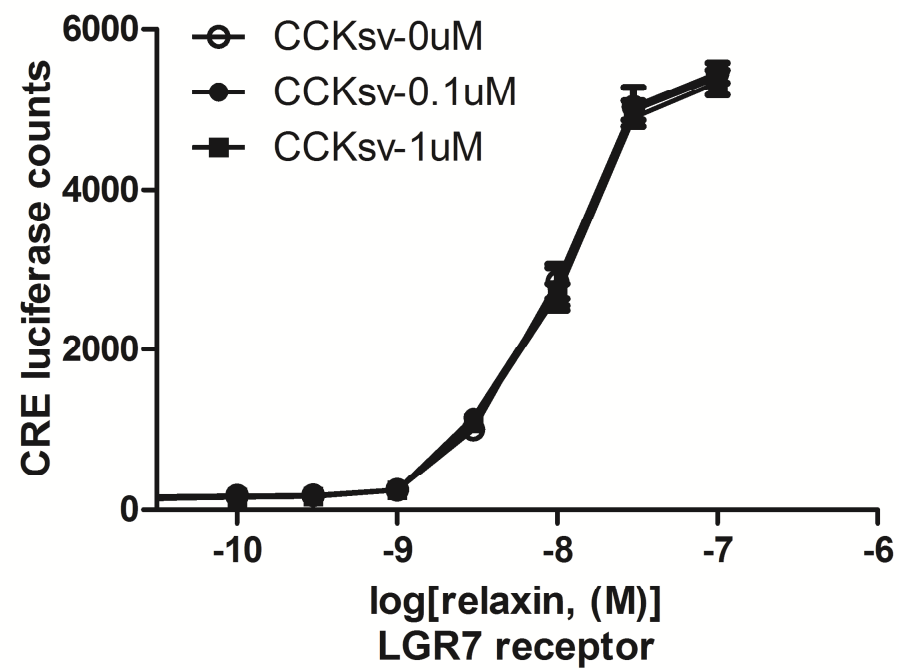

Supplement: Figure S6 — CCKsv cannot alter the CRE-luciferase activity stimulated by relaxin in LGR7-expressing cells. (PDF) [file pone.0064610.s006.pdf]
