# Supplementary material for: Evolution of a Potential Hormone Antagonist following Gene Splicing during Primate Evolution
Source: PLoS One. 2013 May 28;8(5):e64610. doi: 10.1371/journal.pone.0064610 (PMC3665846; doi:10.1371/journal.pone.0064610)
Supplement: Figure S4 — Protein sequence of V5 tagged CCKsv. The V5 tag was inserted into the CCKsv mature region. (PDF) [file pone.0064610.s004.pdf]

Fig. S4

>CCKsv
MNSGVCLCVLMAVLAAGALTQPVPPADPAGSGLQRAEEAPRRQLRVSQRTDGESRAHLGALLARYIQQ
ARKGKNAASPSLTSALVPRLPMLTLFSSASLMGMTSLGRKQAT-

>V5 tagged CCKsv
MNSGVCLCVLMAVLAAGALTQPVPPADPAGSGLQRAEEAPRRQLRVSQRTDGESRAHLGALLARYIQQ
ARK GKNAASPSLTSALVPR GKPIPNPLLGLDST LPMLTLFSSASLMGMTSLG RKQAT-

V5 tagged
