# Supplementary material for: Evolution of a Potential Hormone Antagonist following Gene Splicing during Primate Evolution
Source: PLoS One. 2013 May 28;8(5):e64610. doi: 10.1371/journal.pone.0064610 (PMC3665846; doi:10.1371/journal.pone.0064610)
Supplement: Figure S7 — Alignment of CCKsv read though sequences in primates. The putative poly-adenylation signal is shown in bold and underlined. (PDF) [file pone.0064610.s007.pdf]

```
CLUSTAL X (1.8) multiple sequence alignment                    Fig. S7


Human          GTAAGAATGCTGCCTCCCCATCCCTCACTTCTGCCCTTGTTCCCAGGCTCCCGATGCTGA
Chipanzee      GTAAGAATGCTGCCTCCCCATCCCTCACTTCTGCCCTTGTTCCCAGGCTCCCAATGCTGA
Orangutan      GTAAGAATGCTGCTTCCCCATCCCTCACTTCTGCCCTTGTTCCCAGGCTCCTGATGCTGA
Gibbon         GTAAGAATGCTGCCTCCCCATCCCTCACTTCTGCCCTTGTTCCCAGGCTCCCGATGCTGA
Baboon         GTAGGAATGCTGCCTCCCCATCCCTCACTTCTGCCCTTGTTCCCAGGCTCTGGATGCTGA
Rhesus         GTAAGAATGCTGCCTCCCCATCCCTCACTTCTGCCCTTGTTCCCAGGCTCTGGATGCTGA
Squirrel M     GTAAGAATGC-GCCTCCCCATCCCTCACCTCTGCCCTCCTTCCCAGGCTCC——ATGCGGA
Marmoset       GTAAGAATGC-GCCTCCCCATCCCTCACCTCTGCCCTGGTTCCCAGGCTCC--ATGCAGA
               *** ****** ** ************** ********  ***********   **** **

Human          CCCTCTTCTCTAGCGCTAGCCTGATGGGGATGACCTCTCTCGGTAGGAAACAAGCAACAT
Chipanzee      CCCTCTTCTCTAGCGCTGGCCTGATGGGGATGACCTCTCTCGGTAGGAAACAAGCAACAT
Orangutan      CCCTCTTCTCTAGCCCTGGCCTGATGAAGATGACCTCTCTCGGTAGGAAACAAGCAACAT
Gibbon         CCCTCTTCTCTAGCGCTGGCCTGATGGAGATGACCTCTCTCGGTAGGAAACAAGCAACAT
Baboon         CCCTCTTCTCTAGCGCCGGCCTGATGAAGATGACCTCTCTCGGTAGGAAACAAGCAACAT
Rhesus         CCCTCTTCTCTAGCGCCGGCCTAATGAAGATGACCTCTCTCGGTTGGAAACAAGCAACAT
Squirrel M     CCCTCTTC---AGTGCCGGCCTGATGGAAGTAACCTCTCTGGGTTGGCACCAAGCAACGT
Marmoset       CCCTCTTCTCCAGGGCCGGCCCGATGGAGGTAACCTCTCTCGGTAGGAACCAAGCAACGT
               ********   **  *  ***  ***    * ******** *** ** * ******** *

Human          GATTTCTGGCGGTCCTTTGTAGCAATCTGAGAAGGGGTATGGAGGACTTAATTTAT-AAG
Chipanzee      GGTTTCTGGCGGTCCTTTGTAGCAATCTGAGAAGGGGTATGGAGGACTTAATTTAT-AAG
Orangutan      GGTTTCTGGCAGTCCTTTGTAGCAATCTGAGAAGGGGTATGAAGGACTTAACTTAT-AAG
Gibbon         GGTTTCTGGCCGTCCTTTGTAGCAATCTGAGAAGGGGTATGGAGAACTTAATTTAT-AAG
Baboon         GGTTTCTGGCCGTCCTTTGTAGCAATCTGAGAAGGGGTATGGAGGACTTAATTTAT-AAG
Rhesus         GGTTTCTGGCCGTCCTTTGTAGCAATCTGAGAAGGGGTATGGAGGACTTAATTTAT-AAG
Squirrel M     GGTTTCTGGCCGTCCTTTGTAGCAATCTGAGAAGGGGTACGGAGGACTTAACTTATTAAG
Marmoset       GGTTTCTGGCCGTCCTTTGTAGCAATCTGAGAAGGGGTACGGAGGACTTCATTTAT-AAG
               * ******** **************************** * ** **** * **** ***

Human          TAAGGGGAACCTTCTTGAAAAGCTTTCTTTGAACTAATTTTTAG--CAGTGTTGCTGATT
Chipanzee      TAAGGGGAACCTTCTTGAAAAGCTTTCTTTGAACTAATTTTTAG--CAGTGTTGCTGATT
Orangutan      TAAGGGGAACCTTCTTGAAAAGCTTTCTTTGAACTAATTTTTAG--CAGTGTTGCTGATT
Gibbon         CAAGGGGAACCTTCTTGAAAAGCTTTCTTTGAACTAATTTTTAG--CAGTGTTGCTGATT
Baboon         TAAGGGGAACCTTCTTGAAAAGCTTTCTTTGAACTAATTTTTAG--CAATGTTGCTGATT
Rhesus         TAAGGGGACCCTTCTTGAAAAGCTTTCTTTGAACTAATTTTTAG--CAATGTTGCTGATT
Squirrel M     TAAGGGGGACCTTCTTTAAAAGCTTTCTTTGAACTAATTTTTAGTACAGTGTTGCTGATT
Marmoset       TAAGGGGAGCCTTCCTAAAAAGCTTTCTTTGAACTAATTTTTAGCACAGTGTTGCTTATT
                ******  ***** * ***************************  ** ******* ***

Human          AAGTTCAACACCATTTTACCAAGCCATTAAAAGATAATTATTGAAGCCTAGGAAAGGGTT
```

```
Chipanzee      AAGTTCAACACCATTTTACCAAGCCATTAAAAGATAATTATTGAACCCTAGGAAAGGGTT
Orangutan      AAGTTCAATACCATTTTACCAAGCCATTAAAAGATAATTATTGAATCCTAGGAAAGGGTT
Gibbon         AAGTTCAACACCATTTCACCAAGCCATTAGAAGATAATTATTGAACCCCAGGAAAGGGTT
Baboon         AAGTTCAACACCATTTTACCAAGCCATTAAAAGATAATTATTGAACCCTAG-AAAGGGTT
Rhesus         AAGTTCAACACCATTTTACCAAGCCATTAAAAGATAATTATTGAACCCTAGGAAAGGGTT
Squirrel M     AAATTCAACACAATTTTACCAAGCCAGTAAAAGATAATTATTGAACCTGAGGAGAGGGTT
Marmoset       AAGTTCAACACCATTTTACCAAACCAGTAAAAGATAATAATTGAACCTGAGGAAAGCGTT
               ** ***** ** **** ***** *** ** ******** ****** *  ** * ** ***

Human          CCTAGATACAGTGAATACCCTCTACTGAAAA-CGCAAAGGACCTTGGAGCCACCCAG-AT
Chipanzee      CCTAGATACACTGAATACCCTCTACTGAAAA-CGCAAAGGACCTTGGAGCCACCCAG-AT
Orangutan      CCTAGATACACTGAATACCCTCTACTGAAAAACGCAAAGGACCTGGGAGCCACCCGG-AT
Gibbon         CCTACATACACTGAACACTCTCTACTGAAAA-CGCAAAAGACCT-GGAGCCACCCAG-AT
Baboon         CCTAGATACACTAAATACCCTCTACTAAAAA-CGCAA-GAACCTGGGAGCCACCCAG-AT
Rhesus         CCTAGATACACTAAATACCCTCTACTAAAAA-CGCAAAGAACCTGGGAGCCACCCAG-AT
Squirrel M     CCCAGATACACTAAAAGCCCTCTACTGAAAA-CGCAAAGGACCTGGGAGCCACCCAG-AT
Marmoset       CCCAGATACACCAAAAGCCCTCTACTGAAAA-CGCAAAGGACCTGGGAGCCACCCAGTAT
               ** * *****   **  * ******* **** *****   **** ********** * **

Human          ACCAACCCTTGGAAATCCTCTAGCAGGAAGGGGTCAATGCTGAGAATGCCTGAGATCTGG
Chipanzee      ACCAACCCTTGGAAATCCTCTAGCAGGAAGGGGTCAATGCTGAGAATGCCTGAGATCTGG
Orangutan      ACCAACCGTTGGAAATCCTCTAGCAGGAAGGGGTCAATGCTGAGAATGCCTGAGATCTGG
Gibbon         ACCAACCCTTGGAAATCCTCTAGCAGGAAGGGGTCAATGCTGAGAATGCCTGAGATCTGG
Baboon         ACCAACCCTTGGAAATCCTCTAGCAGGAAGGCGTCAATGCTGA-AATGCCTGAGATCTGG
Rhesus         ACCAACCCTTGGAAATCCTCTAGCAGGAAGGCGTCAATGCTGAGAATGCCTGAGATCTGG
Squirrel M     ACCAACCCTTGGAAATCTTCTAGCAGGAAGGGGTCAGTGCTGAGAATGCCTGAGGTCTGG
Marmoset       ACCAACCCTTGGAAATCCTCTAGCAGGAAGGGGTCAGCGCTGAGAATGCCTGAGGTCTGG
               ******* ********* ************* ****  ***** ********** *****

Human          CTACAACAGTTTA--AAAAAGCAGTGACAATGTGTCCATTTCCACCAAAGTATGTAACAG
Chipanzee      CTACGACAGTTTA--AAACAGCAGTGACAATGTGTCCATTTCCACCAAAGTATGTAACAG
Orangutan      CTACAACAGTTTA--AAAAAGCAGTGACAATGTGTGCATTTCCACCAAAGTATGTAACAG
Gibbon         CTACAACCGTTTA--AAAAAGCAGTGACAATGTGTCCATTTCCACCAAAGTATGTAACAG
Baboon         CTACAACAGTTTA--AAAAAGCAGTGACAATGTGTCCATCTCCACCAAACCATGTAACAG
Rhesus         CTACAACAGTTTA--AGAAAGCAGTGACAATGTGTCCATCTCCACCAAACCATGTAACAG
Squirrel M     CTACAACCGTTTTTTAAAAAGCAATGACAATGTGTCCATTTCCACCAAAGCATGTCACAG
Marmoset       CTACAACCGTTTT--AAACAGCAATGCCAATGTGTCCATTTCCACCAAAGCATGTCACAG
               **** ** ****   * * **** ** ******** *** *********  **** ****

Human          AAGGAAAGAATCAGCAGCAGTTTTGTGTTCTCCTTGCAATGACTTACAAATTATTACCCA
Chipanzee      AAGGAAAGAATCAGCAGCAGTTTTGTGTTCTCCTTGCAATGACTTACAAATGATTACCCA
Orangutan      AAGGAAAGAATCAGCAGCAGTTTTGTGTTCTCCTTGCAATGACTTACAAATGA---CCCA
Gibbon         AAGGAAAGAATCAGCAGCAGTTTTGTGTTCTCCTTGCAATGACTTACAAATGATTACCCA
Baboon         AAGGAAAGAATCAGCAGCAGTTTTGTGTTCTCCTTGCAATGACTTACACATGATTACCCA
```

```
Rhesus          AAGGAAAGAATCAGCAGCAGTTTTGTGTTCTCCTTGCAATGACTTACACATGATTACCCA
Squirrel M      AAGGAAAGAACCAGCAGCAGTTTTGTGTTCTCCTTGCAGTGACTTACAGATGATTACCCA
Marmoset        AAGGAAAGAACCAGCAGCAGTTTTCTGTTCT---TGCAATGACTTAGAAATGATGACCCA
                ********** ************* ******   **** ******* * ** *   ****

Human           ACAAGAGAGGTATTTGTTTTTACTTTACTGTTGCAGAGATGGTCACAGTTGTAGCAAGAG
Chipanzee       ACAAGAGAGGTATTTGTTTTTACTTTACTGTTGCAGAGATGGTCACAGTTGTAGCAAGAG
Orangutan       ATAAGAGAGGTCTTTGTTTTTACTTTGCTGTTGCAGAGATGGTCACAGTTGTAGCAAGAG
Gibbon          ACAAGAGAGGTATTTGTTTTTACTTCACTGTTGCAGAGATGGTCACAGTCGTAGCAAGAG
Baboon          ACAAGAGAGGTATTTGTTTTTACTTTACTGTCGCAGAGATGGTCACAGTTATAGCAAGAG
Rhesus          ACAAGAGAGGTATTTGTTTTTACTTTACTGTCGCAGAGATGGTCACAGTTATAGCAAGAG
Squirrel M      ACAAGAGCTGTATTTGTTTTTCCTTTAGTGTTACAGAGATGGTCTCAGTTGTAGCAAGAG
Marmoset        GCAAGGGCGGCGTTTGTTTTTCCTTTGGTGTTACAG---TGGTCTCAGTTGTGGCAAGAG
                  *** *  *  ********* ***   ***  ***   ***** ****  * *******

Human           CTGAGCAAGTCAGTTTTCTCAGCATCAAAGTCTTTCTCCAGGAATAAGGTGATTCAGCAG
Chipanzee       CTGAGCAAGTCAGTTTTCTCAGCATCAAAGTCTTTCTCCAGGAATAAGGTGATTCAGCAG
Orangutan       CTGAGCAAGTCAGTTTTCTCAGCATCAAAGTCTTTCTCCAGGAATAAGGTGATTCAGCAG
Gibbon          CTGAGCAACTCAGTTTTCTCAGCATGAAAGTCTTCCTCCAGGAATAAGGTGATTCAACAG
Baboon          CTGAGCAAGTCATTTTTCTCAGCATCAAAGTCTTTCTCCAGGAATAAGGTGATTCAGCCC
Rhesus          CTGAGCAAGTCATTTTTCTCAGCATCAAAGTCTTTCTCCAGGAATAAGGTGATTCAGCCC
Squirrel M      CGGAGCAAGTCGGTTTTCTCAGTATCAAAACCTTTCTCCAGGAAAAAGGTGATTCAGGAG
Marmoset        CGGAACAAGTCGGTTTTCTCAGCCCCAAAGCCTTTCTCCAGGAA--------TTCAGCAC
                * ** *** **  *********    ***  *** *********        ****

Human           CTCACTAGCAAAAAAA
Chipanzee       CTCACTAGC
Orangutan       CTCACTAGC
Gibbon          CTCACTAGC
Baboon          CTCACTAGC
Rhesus          CTCACTAGC
Squirrel M      CTCACTAGC
Marmoset        TTCACTAGC
                 ********
```
